# Determinants of quality in the independent and public hospital sectors in England

**DOI:** 10.1093/intqhc/mzaf019

**Published:** 2025-03-05

**Authors:** Harriet Bullen, Vasudha Wattal, Rachel Meacock, Matt Sutton

**Affiliations:** Health Organisation, Policy and Economics, School of Health Sciences, University of Manchester, Oxford Road, Manchester M139PL, United Kingdom; Health Organisation, Policy and Economics, School of Health Sciences, University of Manchester, Oxford Road, Manchester M139PL, United Kingdom; Health Organisation, Policy and Economics, School of Health Sciences, University of Manchester, Oxford Road, Manchester M139PL, United Kingdom; Health Organisation, Policy and Economics, School of Health Sciences, University of Manchester, Oxford Road, Manchester M139PL, United Kingdom

**Keywords:** health care economics and organizations, quality of health care, hospital care, public health, private health

## Abstract

**Background:**

Increasing the use of independent providers has been proposed as a solution to the long waiting times at public hospitals generated by the postpandemic backlog for elective care. However, the profit-maximizing aims of some independent providers may risk cost-cutting behaviours and reduced care quality. Empirical evidence on the extent to which these concerns are borne out in practice is sparse. We aim to examine the quality of acute hospital care provided by the public and independent hospital sectors in England and explore the drivers of variation in quality.

**Methods:**

We construct a unique dataset collating publicly available Care Quality Commission (CQC) quality ratings of independent and public acute hospitals as of December 2022 and 2020. We link these to regional deprivation indices, population estimates, average household disposable incomes, and referral to treatment (RTT) data. We first categorize providers into National Health Service (NHS) and independent hospitals to analyse the association of ownership with quality ratings. To analyse ownership further, we then subcategorize independent hospitals further and consider whether the organization provides NHS-commissioned care. Thus, hospitals were categorized into seven mutually exclusive categories: NHS provider, commissioned charity, commissioned brand, commissioned independent other, noncommissioned charity, noncommissioned brand, and noncommissioned independent other. We use linear and ordered logistic regression models to assess the association of ownership with quality ratings. In supplementary analysis, we examine consistency over time by comparing the effects on 2022 ratings and 2020 ratings.

**Results:**

Of the 283 NHS hospitals, 47.3% (*N* = 134) was rated ‘Good’ and 41.0% (*N* = 116) was rated as ‘Requires Improvement’. Of the 453 independent hospitals, 82.3% (*N* = 373) was rated ‘Good’ and 9.5% (*N* = 43) was rated as ‘Requires Improvement’. On average, independent hospitals had 0.205 (Standard Error [SE] = 0.0581) higher category quality ratings than NHS providers. All types of NHS-commissioned independent sector hospitals had higher average quality ratings than NHS hospitals, as did noncommissioned branded hospitals. Quality ratings were negatively related to the number of different services provided, suggesting that specialization is associated with higher quality.

**Conclusion:**

We find higher quality ratings for independent providers providing NHS-funded care, branded providers, and providers with a narrower range of services. We find no evidence to suggest that outsourced patients will experience lower quality care, although cream-skimming could still be detrimental for NHS services if they are left with a more complex case mix. Overall, our results taken together suggest that the increasing number of NHS patients treated in the independent sector does not experience a worse quality of care, especially if providers specialize in a limited number of services.

## Introduction

The private hospital sector is expanding internationally, with many countries adopting a mixed system with both private and public providers [1]. Understanding the differences in quality across independent and public hospitals is therefore vital to examine if patients are getting the same quality of care regardless of organizational ownership.

For instance, the contracting out of elective care to independent providers in New Zealand was found to increase the number of patients treated while also reducing waiting times in public hospitals [2]. Norway also has a procurement system based on competitive tendering to outsource elective surgery, where private-for-profit firms performed day surgeries at a lower price than public hospitals [3]. The lower cost of day surgeries was speculated to be caused by streamlined care, less teaching responsibilities, the absence of acute services, and a less severe patient mix [3].

Major reforms of the NHS in England in the 2000s enabled independent hospitals to provide NHS-commissioned care [4]. These reforms aimed to enhance healthcare capacity, alleviate waiting times, and address the cancellation of elective care caused by emergency care priorities [5]. The reforms aimed to not only improve quality and efficiency in hospitals but also incentivize competition between hospitals [6]. The NHS has subsequently seen increases in privatization through outsourcing [7], with £9.4 billion spent procuring care for NHS patients from independent sector providers in 2018/19 [8]. The NHS is particularly reliant on independent sector providers for specific procedures, with 20% of cataract procedures and 30% of hip replacements outsourced to independent providers in 2017–18 [8].

This increased reliance on the independent sector has led to concerns regarding the consistency of care quality for NHS patients when services are outsourced to independent providers [9]. Therefore, it is paramount to understand the differences in quality ratings of independent and NHS hospitals.

For-profit independent providers have the organizational goal of profit maximization [10], which is often linked to negative impacts on welfare and quality. When governments outsource public services to for-profit organizations, innovation and quality are theorized to be dominated by cost minimization incentives [11]. Additionally, there could be a risk of cherry-picking patients [12]. Kelly and Stoye [6] found that patients have fewer comorbidities and a slightly lower mean age at independent hospitals, potentially leaving a severe patient mix at public hospitals.

However, there is some evidence of positive impacts of outsourcing care to the independent providers. Kelly and Stoye [6] found that private hospital entry increased the overall volume of hip replacements without impacting quality. Gaynor *et al*. [13] found that the NHS England reforms were successful, slightly reducing mortality without increasing costs.

Quality of care can be defined as the optimization of health benefits from healthcare for a patient’s specific needs and their capacity to access this care [14]. A comprehensive quality measurement strategy involves feedback, monitoring, and evaluation of progress against nationally set goals, with the objective of employing indicators as measurement tools to analyse the success of a strategy [15]. Many methods can be used to measure quality, including staff and patient surveys or observation from an independent regulator.

The existing literature in England has focused on the impact of reforms on specific patient groups or outcomes such as mortality, as opposed to examining service quality more generally. This is similar in the international literature on privatization [2, 3].

The aim of this paper is to compare the quality ratings of public and independent hospitals. This is necessary to understand if increasing outsourced care to the independent sector can reduce waiting times without risking quality. Specifically, our main research question asks: Is ownership associated with quality ratings?

## Methods

### Data

We used publicly available data on provider quality ratings from the CQC, the independent regulator of health and social care in England. Inspections are arranged as needed on a flexible rather than fixed schedule, with the last rating awarded applying until another inspection is complete [16].

We used CQC ratings from two points in time: 2 January 2020 and 1 December 2022. These reflect the latest published ratings as of those dates and do not necessarily indicate the year an inspection was carried out. We downloaded these data on 28 July 2023 [17]. Our sample comprised 314 NHS locations and 839 independent hospitals.

The CQC awards a separate rating to each provider for each service they deliver, in addition to an overall rating for all their services combined. We focus on acute hospital providers in England, for which the data contain ratings on up to 54 different services. The CQC considers five domains when rating a service [18] (see Supplementary Appendix Five Domains).

Each service can be awarded a rating of

- ‘Outstanding’, if the service is exceptionally performing;
- ‘Good’, if the service is meeting all expectations;
- ‘Requires Improvement’, if the CQC have told the service how it needs to improve and is not performing well; or
- ‘Inadequate’, if performance of the service is bad and the CQC has acted against the organization that runs the service [19].

We utilized these overall ratings for each hospital in our analysis.

For each hospital, the CQC dataset provides information on whether it is NHS run or independent, the type of services provided, the publication date of the rating, and where it is located including NHS region, local authority, and postcode. For independent providers, information on whether a hospital is registered as a brand or charity is also provided. This information was derived from the variables Location Type, Service/Population Group, Location Region, Location Local Authority, Location Post Code, and Brand ID. We classified the remaining hospitals that were not a charity or had brand affiliation as ‘independent other’.

We restricted our sample based on the following criteria: the classification of location type, primary inspection category, domain, and service or population group. Specifically, within Location Type, we selected ‘Independent Healthcare Org’ and ‘NHS Healthcare Organisation’. From Location Primary Inspection Category, we selected four categories ‘Acute Hospital – Independent non-specialist’, ‘Acute Hospital – Independent specialist’, ‘Acute Hospital – NHS non-specialist’, and ‘Acute Hospital – NHS specialist’. Within Domain, we selected ‘Overall’ to obtain the latest overall rating, and within Service/Population Group, we selected ‘Overall’. This restriction approach ensures that we have only one observation for every hospital that is NHS or independently owned. After restricting the dataset, we had a total of 314 NHS hospitals and 839 independent hospitals.

The identification of a charity required the use of an additional CQC dataset care directory with filters [17]. We obtained data on socioeconomic indicators from the Office for National Statistics (ONS) deprivation indices, specifically the Index of Multiple Deprivation, aggregated at Clinical Commissioning Group (CCG) level [20]. We also obtained age [21] proportions and gender [22] percentages from the ONS aggregated at the local authority level. We also use latest available estimates of population size captured at the CCG level [23] and a regional measure of average household disposable income from ONS Gross Domestic Household Income (GDHI) [24]. Although Integrated Care Boards replaced CCGs on 1 July 2022, the data still attribute providers to CCGs using the Location ONS Postcode Directory CCG Code, and so we use CCGs as our population estimates and Index of Multiple Deprivation (IMD) from the ONS are per CCG.

We also used RTT waiting time data from the NHS to identify hospitals that provide NHS-commissioned care [25]. These RTT data are collected to monitor waiting times and cover all referrals for NHS-funded care to both NHS and independent providers. We classify independent hospitals as providing some NHS-funded care if they had one or more referral recorded with a clock start date in January, June, or December of that year.

### Analysis

We assigned a score to each of the four rating categories, where Inadequate corresponds to a score of 1, Requires Improvement a score of 2, Good a score of 3, and Outstanding a score of 4. We used linear regression in the main analysis to assess the association of ownership type (NHS run/independent) and number of services on overall quality ratings.

Initially, we categorized hospitals into two groups: NHS and independent hospitals. We subdivided our hospital categories further depending on the type of independent hospital and whether they provide any amount of NHS-commissioned care. This resulted in seven mutually exclusive categories: NHS provider, commissioned charity, commissioned brand, commissioned independent other, noncommissioned charity, noncommissioned brand, and noncommissioned independent other.

We included the following additional covariates: region, level of competition measured by the number of hospitals in each CCG, size of population, and level of deprivation. This enables us to examine whether the estimated differences in quality between ownership types are influenced by variation in regional demand, competition, and socioeconomic indicators.

We undertake our main analyses on the latest ratings as of 2022. In a supplementary analysis, we compare 2020 and 2022 as a robustness check. To ensure that these comparisons were not driven by the additional hospitals that had recently entered the market, we restricted our sample to a balanced panel of hospitals meeting our criteria in both 2020 and 2022 for this robustness check.

Additionally, the December 2022 contains ratings given during the coronavirus disease (COVID-19) pandemic period. The pandemic put considerable strain on hospitals, and so the ratings then may reflect differently on hospital quality. To examine this impact, we repeated the analysis on the January 2020 ratings given before the COVID-19 pandemic.

Using a common sample of hospitals, we pooled the data from 2020 and 2022 into one regression model and interacted every variable with a year indicator to test whether the rating of each type of organization had changed over time. This was to test whether the rating of each type of organization had changed over time.

In further supplementary analysis, we repeat our main analysis using ordered logistic regression models to assess robustness to the assumption of a linear relationship between covariates and quality rating imposed by treating the scores as cardinal rather than their original ordinal form.

As the CQC has a flexible inspection schedule, it may introduce selection bias if some hospitals are rated more frequently than others. To assess the importance of this issue for our analysis, we have obtained a variable representing the time since the last CQC inspection. We included this variable into our regression models as a sensitivity analysis.

The overall rating is aggregated over five rating domains. To ensure that no specific aspects of the five rating domains are lost, we ran our regressions on the separate rating domains. However, some hospitals do not have a separate rating for all the five domains.

There are likely to be differences in case mix between independent and NHS providers [6]. To examine whether this affected the comparison, in a supplementary analysis, we excluded 154 of the 314 NHS providers with a Type 1 A&E department. Type 1 A&Es are 24-h consultant led with designated accommodation for accident and emergency patients and resuscitation facilities [26] and will result in these providers having more complex patient case mix.

In additional sensitivity analysis, we ran the analysis restricted to three specific services that are provided by the highest numbers of NHS and independent hospitals: surgery, outpatients, and medical care (including older people’s care). This was to account for differences in case mix at NHS and independent hospitals.

All analyses were undertaken using Stata v17 and used ‘robust’ standard errors.

## Results

### Descriptive statistics

**Figure 1:**
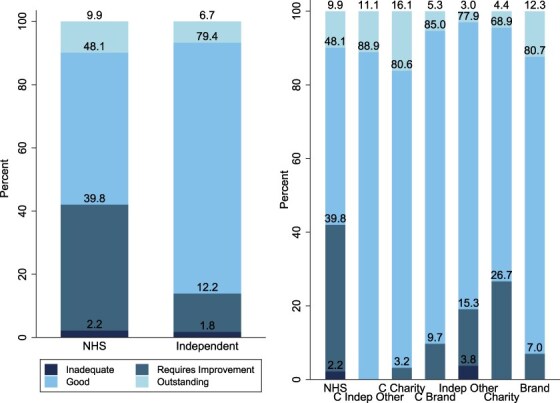
Distribution of ratings across hospital categories.

Notes: This shows the distribution of rating across the different hospital categories. In the left panel, hospitals are split into two categories (NHS and independent). In the right panel, the classification of independent providers is more refined, examining seven categories of provider in total. Percentages are shown on the stacked bars.

Of the 314 NHS hospitals in our sample, 9.9% (*N* = 31) was rated ‘Outstanding’ in 2022, 48.1% (*N* = 151) ‘Good’, 39.8% (*N* = 125) as ‘Requires Improvement’, and 2.2% (*N* = 7) as ‘Inadequate’ (Fig. 1). Of the 839 independent hospitals, 6.7% (*N* = 56) was rated ‘Outstanding’, 79.4% (*N* = 666) ‘Good’, 12.2% (*N* = 102) as ‘Requires Improvement’, and 1.8% (*N* = 15) as ‘Inadequate’ (Fig. 1, full descriptive statistics are in the Supplementary Appendix Tables S1 and S2).

With NHS-funded care, independent other had the highest percentage of ‘Good’ ratings and charity has the highest percentage of ‘Outstanding’ ratings. Additionally, noncommissioned charity has the largest proportion of inadequate, whereas commissioned independent other had no providers rated as ‘Inadequate’.

**Figure 2:**
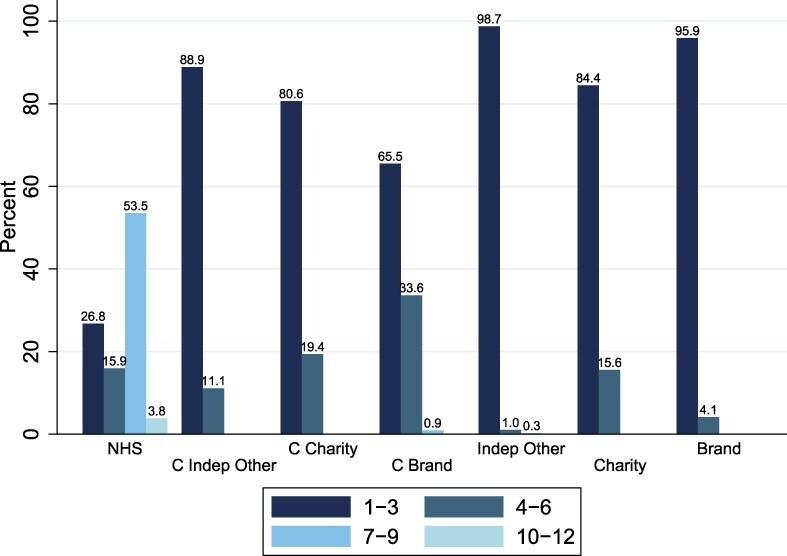
Distribution of number of services provided across hospital categories in 2022.

Notes: This figure shows the number of different services provided across the different hospital categories. The percentage within each hospital category is shown on the bars.

In terms of service provision (Fig. 2), NHS hospitals provided more types of services, with 53.5% providing seven to nine different services. Across all the six independent categories, the majority of hospitals provide one to three different services, suggesting specialization (Fig. 2).

### Main analysis

On average, ratings of independent hospitals were 0.166 (SE = 0.0581) higher than the reference group of NHS hospitals on a 1–4 categorical scale (Table 1, Column 1). Each additional service that a hospital provided was associated with a 0.0203 (SE = 0.00964) lower rating.

**Table 1.** Results of linear regression of quality ratings on ownership type, 2022

	(1) NHS vs independent	(2) Seven provider categories	(3) With covariates
Independent	0.166** (0.0581)		
Commissioned independent other		0.435*** (0.121)	0.415** (0.131)
Commissioned charity		0.361*** (0.0856)	0.379*** (0.0844)
Commissioned brand		0.253*** (0.0593)	0.266*** (0.0578)
Independent other		−0.0207 (0.0672)	−0.0409 (0.0671)
Charity		−0.0401 (0.100)	−0.0412 (0.0956)
Brand		0.216*** (0.0691)	0.200** (0.0683)
Number of services	−0.0203* (0.00964)	−0.0337* (0.0104)	−0.0338*** (0.0102)
Observations	1153	1153	1153

Reference ownership type is the NHS provider in all models. Coefficients from linear regression of overall quality rating scored as follows: 4, ‘Outstanding’; 3, ‘Good’; 2, ‘Requires Improvement’; 1, ‘Inadequate’. Robust standard errors are given in parentheses. Model 3 includes area deprivation, population size, competition, region, proportion of population aged 35–64, 65–84, 85+ years, and percentage of males.

* *P* < .05.

** *P* < .01.

*** *P* < .001.

On average, hospitals categorized as ‘commissioned Independent other’ had ratings 0.435 (SE = 0.121) higher than the reference group of NHS hospitals (Table 1, Column 2), while commissioned charities had ratings 0.361 (SE = 0.0856) higher and commissioned brand providers 0.253 (SE = 0.0593) higher ratings than NHS hospitals.

Ratings of ‘independent other’ and independent charity providers did not significantly differ from those of NHS hospitals. However, branded hospitals not providing NHS-commissioned care had ratings 0.216 (SE = 0.0691) higher on average in comparison to NHS hospitals. In the model with seven provider types, each additional service a hospital provided was associated with 0.0337 (SE = 0.0104) lower ratings.

The estimated differences in quality between ownership types were not substantially influenced by the addition of covariates measuring regional demand, competition, and socioeconomic indicators (Table 1, Column 3; full regression results in the Supplementary Appendix Table S3).

### Supplementary analysis

Restricting our sample to a balanced panel of hospitals providing acute services in both 2020 and 2022 resulted in 283 (90.13% of original 314) NHS locations and 453 (53.99% of original 839) independent hospitals, indicating that there has been a large increase in market entry from private providers between 2020 and 2022. The 2020 analysis to check consistency revealed intriguing results (Table 2). In the 2020 simple model, where hospitals are split up into either NHS or independent (Table 2, Column 1), there was no significant association between ownership type and ratings. This contrasts with the 2022 model where we find that independent hospitals have significantly higher ratings (Table 2, Column 3), suggesting that among the same group of providers, a quality difference has emerged between 2020 and 2022.

**Table 2.** Comparison of linear regressions of quality ratings on ownership type, 2020 and 2022.

	(1) 2020 NHS vs independent	(2) 2020 seven provider categories	(3) 2020 NHS vs independent	(3) 2022 seven provider categories
Independent	0.0828		0.205*** (0.0581)	
Commissioned independent other		0.162* (0.0635)		0.335** (0.117)
Commissioned charity		0.242* (0.106)		0.369*** (0.0926)
Commissioned brand		0.0418 (0.0705)		0.207*** (0.0602)
Independent other		−0.0403 (0.0777)		0.0626 (0.0719)
Charity		0.165 (0.0917)		0.172 (0.111)
Brand		0.230** (0.0785)		0.282*** (0.0736)
Number of services	−0.0393*** (0.0108)	−0.0421*** (0.0114)	−0.0279** (0.00994)	−0.0326** (0.0107)
Observations	736	736	736	736

Reference ownership type is the NHS provider in all models. Coefficients from linear regression of overall quality rating scored as follows: 4, ‘Outstanding’; 3, ‘Good’ 2, ‘Requires Improvement’ 1, ‘Inadequate’. Robust standard errors are given in parentheses. Models 1 and 2 are the 2020 model estimates. Models 3 and 4 are the 2022 model estimates.

* *P* < .05

** *P* < .01

*** *P* < .001

This seems to be driven by the independent providers who undertake NHS-commissioned care and to a lesser extent branded independent providers who are not NHS commissioned (Table 2, Columns 2 versus 4). Independent other and charity providers not undertaking NHS-commissioned care do not have significantly different ratings than NHS providers in either 2020 or 2022. We find that the number of services provided is negatively associated with quality ratings in both 2020 and 2022.

We investigated if there was any significant association of the ratings in 2020 and 2022 (Supplementary Appendix Table S4) by introducing a year interaction term. Although the gaps between NHS hospitals and other ownership types became wider from 2020 to 2022, these changes were not statistically significant.

The results from the ordered logistic regression model (Table 3) are consistent with our main analyses. The probability of having a higher rating was significantly higher for the following provider types: commissioned independent other [odds ratio (OR) = 4.48, 95% Confidence Interval (CI) (1.628, 12.315)], commissioned charity [OR = 5.24, 95% CI (2.316, 11.840)], commissioned brand [OR = 2.54, 95% CI (1.517, 4.259)], and noncommissioned brand [OR = 3.62, 95% CI (1.907, 6.860)], compared to the reference group of NHS hospitals. We again find a negative association between quality ratings and the number of services provided [OR = 0.88, 95% CI (0.807, 0.952)], suggesting that specialization is associated with higher quality.

**Table 3.** Ordered logistic regression of quality ratings on ownership type, 2022.

Categories	Ratings
Commissioned independent other	4.79^**^ [1.696, 13.523]
Commissioned charity	5.61^***^ [2.469, 12.731]
Commissioned brand	2.60^***^ [1.567, 4.326]
Independent other	1.16 [0.687, 1.942]
Charity	0.98 [0.463, 2.076]
Brand	3.31^***^ [1.855, 5.898]
Number of services	0.88^**^ [0.819, 0.956]
Cut point 1	−4.07 [−4.784, −3.364]
Cut point 2	−1.30 [−1.853, −0.739]
Cut point 3	2.84 [2.208, 3.469]
Number of observations	1153

Coefficients are ORs. 95% confidence intervals are shown in [.].The cuts are the points that define the different levels or categories of the outcome variable, allowing the model to predict the probability of an observation belonging to each category. The model specification is the same as Model (2) in Table 1.

**P < .01

***P < .001

The investigation into any selection bias (Supplementary Appendix Table S5) showed that allowing for the time since the last inspections does not change the pattern of results.

When investigating the different rating domains (Supplementary Appendix Table S6), we found that the differences in quality are similar to the pattern for overall quality for all domains and more pronounced for responsive, safe, and well-led. Caring and effective could be less pronounced because of the smaller sample size.

Restricting our sample to the (49%) of 314 NHS hospitals without a Type 1 A&E department, in the 2022 simple model (Supplementary Appendix Table S7), we found a similar pattern of results, but the magnitude of the difference between NHS and independent providers coefficients reduces. Hence, some of the differences we are finding in the main analysis are driven by NHS hospitals, with a Type 1 A&E being on average a lower quality than NHS hospitals without a Type 1 A&E department.

Restricting our analysis to three services that are provided by the highest numbers of NHS and independent hospitals (Supplementary Appendix Table S8), we find similar patterns of results with charity-owned providers now having significantly higher quality ratings than NHS hospitals. The results for hospitals providing outpatients are consistent with our main results. For surgery, the pattern of the results is similar; however, they are no longer statistically significant. For medical care, the commissioned hospital categories are no longer significant, but the results follow a similar pattern.

## Discussion

### Statement of principal findings

We examined the relationship between ownership type and quality ratings among acute hospitals in England, finding that independent providers receive higher quality ratings on average than NHS hospitals. When examining provider type more finely, all types of independent providers who undertake NHS-commissioned care, as well as branded independent providers who do not, had significantly higher quality ratings than their NHS counterparts. We detect a negative relationship between the number of different services provided and overall quality rating, suggesting that specialization is associated with higher quality care. Taken together, these results suggest that the growing number of NHS patients treated in independent hospitals will not experience worse quality care as a result of increased outsourcing, counter to previous concerns [9], particularly if outsourced to providers specializing in the provision of a limited number of services.

### Strengths and limitations

Our findings were robust across the use of linear or ordered logistic regression models and the addition of indicators measuring demand, competition, and socioeconomic indicators. Additionally, we restricted the analysis to a balanced sample of hospitals providing acute services in both 2020 and 2022 to examine how quality differences have changed over time within the same sample of NHS and independent providers as NHS outsourcing has expanded. Furthermore, our categorization allows for a clear and detailed interpretation of different ownership types and their association with quality ratings.

However, there are some limitations. The CQC does not inspect every ward or service in every inspection unless there are exceptional circumstances, instead of inspecting a random sample of wards or select wards based on factors such as quality and risk [27]. As a result, the ratings may not have considered all areas of care. Additionally, as the CQC started compiling ratings from October 2014, if an inspection was before that date and not thereafter, it will not be included in our dataset. Additionally, we had to use lagged values for IMD, GDHI, and population in our 2022 analyses as the latest data had not yet been released by the ONS.

The December 2022 CQC data contain ratings given during the COVID-19 pandemic period. The pandemic put considerable strain on hospitals, and so the ratings then may not be fully representative of hospital quality.

Our results may not necessarily be generalizable to other types of healthcare providers or hospitals with different healthcare systems, and thus similar work should be done on other healthcare systems and sectors.

### Interpretation within the context of the wider literature

There is evidence that patients in many countries choose hospitals with higher quality ratings, including the Netherlands [28], USA [29], and Germany [30]. Among providers not commissioned to provide NHS care, we found branded providers to be the only category associated with significantly higher quality ratings than NHS hospitals. This may therefore be driven by the importance of brand image in the self-payer market, with branded providers striving to achieve high quality ratings to maintain their market share.

Our finding that the number of different services provided is negatively associated with overall quality is consistent with evidence from Germany, showing that efficiency is positively associated with medical specializations [31]. This result could also suggest that the NHS does not benefit from economies of scale or economies of scope when proving too many services. Freeman *et al*. [32] investigate volume-cost spillovers across elective and emergency care and found a negative spillover as an increased volume of elective care increases the cost of emergency care. This opposes the literature that large healthcare facilities can benefit from efficiency and cost reductions, for instance, Carey *et al*. [33] found that shifting output from single service hospitals to general hospitals, there are potential economies of scale to be exploited. However, while independent providers can choose to focus on specific elective services only, the NHS must provide a full spectrum of emergency care as well. Opportunities for specialization within the NHS may therefore be more limited.

Moreover, there is concern that when public services are outsourced to for-profit organizations, incentives to minimize costs may overshadow quality [11]. However, we do not find any evidence that the hospitals to which the NHS outsourced care were lower quality than NHS hospitals. This aligns with the literature, suggesting that for specific services and populations, the independent sector is sometimes associated with positive outcomes [34–36].

Additionally, many NHS clinicians also provide care in the independent sector [37, 38]. While this may reduce staff input to the NHS, it could also help protect quality in the independent sector. NHS clinicians working in the independent sector could soften the profit maximization aim [10] and protect the altruism associated with the public sector [39] by bringing their NHS values and standards of care. Therefore, instead of the profit motive dominating and reducing quality, involvement of NHS clinicians could counteract this effect and protect quality.

### Implications for policy, practice, and research

We find no evidence that outsourced patients will experience lower quality care, and hence continued outsourcing of care to the independent sector may be beneficial to the NHS during a time of high waiting times. However, future research must evaluate the overall impact of increased outsourcing, examining the implications for the NHS in the round. There has not been a comprehensive evaluation of the wider healthcare system following high volumes of low-complexity procedures shifting to the independent sector; therefore, there is an urgent need to address this gap [40].

The potential for independent providers to specialize in less complex services and provide care to less complex patients may lead to a concentration of more complex patients being treated in NHS hospitals. Policymakers should be mindful that any cream-skimming practices by the independent sector may be detrimental for NHS providers if they are left to treat only the most complex and high-cost patients.

Currently, the independent sector specializes in a narrower range of less complex services, and we find no evidence they are delivering worse quality of care. However, if the independent sector continues to expand to provide a more complex range of services, this must be monitored to ensure that they can care for more complex patients.

## Conclusion

We find higher quality ratings for independent providers providing NHS-funded care, branded providers, and providers with a narrower range of services. We find no evidence to suggest that outsourced patients will experience lower quality care, although cream-skimming could still be detrimental for NHS services if they are left with a more complex case mix. Overall, our results taken together suggest that the increasing number of NHS patients treated in the independent sector does not experience a worse quality of care, especially if providers specialize in a limited number of services.

## Supplementary Material

mzaf019_Supp

## Data Availability

The data underlying this article were accessed from the CQC at https://www.cqc.org.uk/about-us/transparency/using-cqc-data [1].
