# Supplementary material for: Determinants of quality in the independent and public hospital sectors in England
Source: Int J Qual Health Care. 2025 Mar 5;37(1):mzaf019. doi: 10.1093/intqhc/mzaf019 (PMC11932141; doi:10.1093/intqhc/mzaf019)
Supplement: mzaf019_Supp [file mzaf019_supp.zip › suppl_data/Appendix.docx]

**Determinants of quality in the independent and public hospital sectors in England**

**APPENDIX**

**Contents List**

1. **Five Domains: The five domains that the CQC considers when rating a service**
2. **Table 1: Distribution of Ratings across hospital categories**
3. **Table 2: Descriptives for additional covariates**
4. **Table 3: Linear regression with all the explored covariates**
5. **Table 4: Linear regression with a year indicator interaction with hospital categories**
6. **Table 5: Linear regression with months elapsed since the latest rating**
7. **Table 6: Linear regression separated by rating domain**
8. **Table 7: Linear regression removing NHS hospitals with a Type 1 A&E**
9. **Table 8: Linear regression restricted by service type**

**Five Domains: The five domains that the CQC considers when rating a service [1]**

- *Safe*: Are you protected from abuse and avoidable harm?
- *Effective:* Does your care, treatment and support achieve good results and help you maintain your quality of life, and is it based on the best available evidence?
- *Caring:* Do staff involve you and treat you with compassion, kindness, dignity, and respect?
- *Responsive:* Are services organised so that they can meet your needs?
- *Well-led:* Does the leadership of the organisation make sure that it’s providing high-quality care that’s based around your needs? And does it encourage learning and innovation and promote an open and fair culture?’

**Table 1: Distribution of Ratings across hospital categories**

| **Categories** | **Ratings** | | | | |
| --- | --- | --- | --- | --- | --- |
|  | **Inadequate** | **Requires Improvement** | **Good** | **Outstanding** | **Total** |
| **NHS** | 7  (2.23) | 125  (39.81) | 151  (48.09) | 31  (9.87) | 314 |
| **Independent** | 15  (1.79) | 102  (12.16) | 666  (79.38) | 56  (6.67) | 839 |
| **Commissioned Independent Other** | 0 | 0 | 8  (88.89) | 1  (11.11) | 9 |
| **Commissioned Charity** | 0 | 1  (3.23) | 25  (80.65) | 5  (16.13) | 31 |
| **Commissioned Brand** | 0 | 11  (9.73) | 96  (84.96) | 6  (5.31) | 113 |
| **Independent other** | 15  (3.77) | 61  (15.33) | 310  (77.89) | 12  (3.02) | 398 |
| **Charity** | 0 | 12  (26.67) | 31  (68.89) | 2  (4.44) | 45 |
| **Brand** | 0 | 17  (7.00) | 196  (80.66) | 30  (12.35) | 243 |

Notes: The table shows the frequency of ratings across NHS and Independent. The frequency of ratings is also shown across the different independent hospital categories. Percentages are in parentheses. The percentages are the proportion of each rating in relation to the total frequency within its respective category.

**Table 2: Descriptives for additional covariates**

| **Covariate** | **Mean** | **SD** | **Variance** | **P50** | **Min** | **Max** | **Range** |
| --- | --- | --- | --- | --- | --- | --- | --- |
| **Population size 2021** | 533491.9 | 444920.3 | 1.98e+11 | 348072.5 | 114496 | 2111469 | 1996973 |
| **Deprivation** | 22.57 | 8.25 | 68.00 | 21.73 | 9.18 | 52.14 | 42.96 |
| **Number of Services provided** | 2.81 | 2.65 | 7.05 | 1 | 1 | 12 | 11 |
| **Competition** | 30.55 | 38.17 | 1456.92 | 17 | 1 | 132 | 131 |
| **Proportion aged 35 – 64 years** | 0.39 | 0.019 | 0.0004 | 0.39 | 0.33 | 0.44 | 0.11 |
| **Proportion aged 65 – 84 years** | 0.15 | 0.04 | 0.002 | 0.15 | 0.05 | 0.25 | 0.20 |
| **Proportion aged 85+** | 0.023 | 0.007 | 0.0005 | 0.024 | 0.007 | 0.043 | 0.036 |
| **Male proportion** | 48.27 | 0.63 | 0.40 | 48.28 | 46.19 | 49.976 | 3.78 |

Notes: The table presents descriptive statistics for the additional covariates we considered in the analysis. The covariates include Population size, deprivation, number of services provided, competition, proportion of those aged 35 - 64, 65 - 84, 85+, and percentage of Males.

**Table 3: Linear regression with all the explored covariates**

|  | **(1)**  **NHS vs independent** | **(2)**  **Seven provider categories** | **(3)**  **With Covariates** |
| --- | --- | --- | --- |
| **Independent** | 0.166**  (0.0581) |  |  |
| **Commissioned Independent Other** |  | 0.435***  (0.121) | 0.415**  (0.131) |
| **Commissioned Charity** |  | 0.361***  (0.0856) | 0.379***  (0.0844) |
| **Commissioned Brand** |  | 0.253***  (0.0593) | 0.266***  (0.0578) |
| **Independent other** |  | -0.0207  (0.0672) | -0.0409  (0.0671) |
| **Charity** |  | -0.0401  (0.100) | -0.0412  (0.0956) |
| **Brand** |  | 0.216***  (0.0691) | 0.200**  (0.0683) |
| **Deprivation** |  |  | -0.00588  (0.00368) |
| **Population size** |  |  | -0.0889  (0.0579) |
| **Competition** |  |  | 0.00194*  (0.000829) |
| ***Region (Reference: London)*** |  |  |  |
| **East of England** |  |  | 0.0359  (0.103) |
| **East Midlands** |  |  | 0.0697  (0.121) |
| **North East** |  |  | 0.310*  (0.140) |
| **North West** |  |  | 0.131  (0.120) |
| **South East** |  |  | 0.155  (0.0850) |
| **South West** |  |  | 0.0412  (0.116) |
| **West Midlands** |  |  | -0.0430  (0.114) |
| **Yorkshire and the Humber** |  |  | -0.00540  (0.124) |
| **Proportion aged 35 – 64 years** |  |  | -0.809  (1.339) |
| **Proportion aged 65 – 84 years** |  |  | 0.327  (1.447) |
| **Proportion aged 85+** |  |  | -7.905  (9.238) |
| **Proportion male** |  |  | -0.0773*  (0.0361) |
| **Number of services provided** | -0.0203*  (0.00964) | -0.0337*  (0.0104) | -0.0338***  (0.0102) |
| **Observations** | 1153 | 1153 | 1153 |

Notes: Reference ownership type is NHS provider in all models. Coefficients from linear regression of overall quality rating scored as “Outstanding”=4, “Good”=3 ”Requires Improvement”=2 “Inadequate =1”. The reference group for region in model three is London. Robust Standard errors in parentheses. * p<0.05, ** p<0.01, *** p<0.001. Model 3 includes deprivation, population, competition, region, proportion of those aged 35 - 64, 65 - 84, 85+, and percentage of males.

**Table 4: Linear regression with a year indicator interaction with hospital categories**

|  | **(1)**  **Seven provider categories** |
| --- | --- |
| **Commissioned Independent Other** | 0.185***  (0.0524) |
| **Commissioned Charity** | 0.259*  (0.105) |
| **Commissioned Brand** | 0.0581  (0.0656) |
| **Independent other** | -0.0158  (0.0668) |
| **Charity** | 0.189*  (0.0825) |
| **Brand** | 0.254***  (0.0674) |
| **2022** | 0.00353  (0.0561) |
| **Commissioned Independent Other**  **# 2022** | 0.133  (0.119) |
| **Commissioned Charity**  **# 2022** | 0.0952  (0.133) |
| **Commissioned Brand**  **# 2022** | 0.136  (0.0817) |
| **Independent Other**  **# 2022** | 0.0569  (0.0779) |
| **Charity**  **# 2022** | -0.0353  (0.131) |
| **Brand**  **# 2022** | 0.00744  (0.0783) |
| **Number of services provided** | -0.0371***  (0.00781) |
| **Observations** | 1472 |

Notes: Reference ownership type is NHS provider. # denotes the interaction terms. Robust Standard errors in parentheses. * p<0.05, ** p<0.01, *** p<0.001

**Table 5: Linear regression with months elapsed since the latest rating**

|  | **(1)**  **NHS vs independent** | **(2)**  **Seven provider categories** | **(3)**  **With Covariates** |
| --- | --- | --- | --- |
| **Independent** | 0.256***  (0.0581) |  |  |
| **Commissioned Independent Other** |  | 0.454***  (0.0851) | 0.434**  (0.143) |
| **Commissioned Charity** |  | 0.315***  (0.0851) | 0.333***  (0.0845) |
| **Commissioned Brand** |  | 0.303***  (0.0597) | 0.316***  (0.0584) |
| **Independent other** |  | 0.102  (0.0685) | 0.0786  (0.0684) |
| **Charity** |  | 0.0896  (0.0963) | 0.0848  (0.0924) |
| **Brand** |  | 0.333***  (0.0703) | 0.314***  (0.0691) |
| **Number of Services provided**  **Months Elapsed** | -0.0122  (0.00933)  0.0054***  (0.0007) | -0.0214*  (0.0104)  0.0047***  (0.0007) | -0.0219*  (0.0102)  0.00461***  (0.000716) |
| **Observations** | 1153 | 1153 | 1153 |

Notes: Reference ownership type is NHS provider in all models. Coefficients from linear regression of overall quality rating scored as “Outstanding”=4, “Good”=3 ”Requires Improvement”=2 “Inadequate =1”. Robust Standard errors in parentheses. * p<0.05, ** p<0.01, *** p<0.001. Model 3 includes area deprivation, population size, competition, region, proportion of population aged 35 - 64, 65 - 84, 85+, and percentage of males. The model now includes a variable for the number of months since the latest inspection.

**Table 6: Linear regression separated by rating domain**

|  | **(1)**  **Caring** | **(2)**  **Effective** | **(3)**  **Responsive** | **(4)**  **Safe** | **(5)**  **Well-led** | **(6)**  **Overall** |
| --- | --- | --- | --- | --- | --- | --- |
| **Commissioned Independent Other** | 0.158  (0.126) | -0.0641 (0.0944) | 0.177*** (0.0476) | 0.391*** (0.0495) | 0.380* (0.147) | 0.435***  (0.121) |
| **Commissioned Charity** | 0.0428  (0.0722) | 0.0414 (0.0575) | 0.316*** (0.0711) | 0.398*** (0.0667) | 0.365*** (0.105) | 0.361***  (0.0856) |
| **Commissioned Brand** | -0.0416  (0.0382) | 0.0854 (0.0475) | 0.258*** (0.0498) | 0.225*** (0.0556) | 0.204*** (0.0597) | 0.253***  (0.0593) |
| **Independent other** | -0.0576 (0.0393) | -0.131* (0.0585) | 0.106  (0.0564) | 0.0317 (0.0617) | -0.101 (0.0700) | -0.0207  (0.0672) |
| **Charity** | -0.0238  (0.0591) | 0.0220 (0.0718) | -0.129 (0.104) | -0.00260 (0.0933) | -0.0581 (0.108) | -0.0401  (0.100) |
| **Brand** | -0.0238 (0.0425) | 0.082 (0.0640) | 0.189** (0.0574) | 0.197*** (0.0611) | 0.200**  (0.0713) | 0.216***  (0.0691) |
| **Number of Services provided** | 0.00909 (0.00619) | -0.0401*** (0.00867) | -0.0313* (0.00935) | -0.0540*** (0.00911) | -0.0147 (0.0107) | -0.0337*  (0.0104) |
| **Observations** | 1116 | 766 | 1142 | 1151 | 1151 | 1153 |

Notes: Reference ownership type is NHS provider in all models. Coefficients from linear regression of overall quality rating scored as “Outstanding”=4, “Good”=3 ”Requires Improvement”=2 “Inadequate =1”. Robust Standard errors in parentheses. * p<0.05, ** p<0.01, *** p<0.001.

**Table 7: Linear regression removing NHS hospitals with a Type 1 A&E**

|  | **(1)**  **NHS vs independent** | **(2)**  **Seven provider categories** | **(3)**  **With covariates** | **(4)**  **Main analysis** |
| --- | --- | --- | --- | --- |
| **Independent** | 0.112  (0.0596) |  |  |  |
| **Commissioned Independent Other** |  | 0.381**  (0.123) | 0.384**  (0.132) | 0.435***  (0.121) |
| **Commissioned Charity** |  | 0.293***  (0.0879) | 0.324***  (0.0870) | 0.361***  (0.0856) |
| **Commissioned Brand** |  | 0.188**  (0.0630) | 0.204***  (0.0616) | 0.253***  (0.0593) |
| **Independent other** |  | -0.0528  (0.0681) | -0.0744  (0.0680) | -0.0207  (0.0672) |
| **Charity** |  | -0.0800  (0.100) | -0.0759  (0.0963) | -0.0401  (0.100) |
| **Brand** |  | 0.182***  (0.0698) | 0.168*  (0.0693) | 0.216***  (0.0691) |
| **Number of Services provided** | 0.00679  (0.0126) | -0.0128  (0.0141) | -0.0158  (0.0140) | -0.0337*  (0.0104) |
| **Observations** | 999 | 999 | 999 | 1153 |

Notes: Reference ownership type is NHS provider in all models. Coefficients from linear regression of overall quality rating scored as “Outstanding”=4, “Good”=3 ”Requires Improvement”=2 “Inadequate =1”. Robust Standard errors in parentheses. * p<0.05, ** p<0.01, *** p<0.001.

**Table 8: Results for specific service types**

|  | **(1)**  **Surgery** | **(2)**  **Outpatients** | **(3)**  **Medical care (including older people’s care)** |
| --- | --- | --- | --- |
| **Commissioned Independent Other** | 0.190  (0.135)  [13] | 0.501*  (0.203)  [5] | 0.109  (0.0673)  [4] |
| **Commissioned Charity** | 0.141  (0.102)  [33] | 0.305***  (0.0727)  [5] | 0.0273  (0.212)  [11] |
| **Commissioned Brand** | 0.0239  (0.0725)  [142] | 0.274***  (0.0793)  [63] | 0.121  (0.0656)  [46] |
| **Independent other** | -0.298**  (0.0976)  [131] | 0.206  (0.124)  [42] | -0.0307  (0.215)  [13] |
| **Charity** | -0.0894  (0.128)  [8] | 0.305***  (0.0754)  [5] | 0.149**  (0.0563)  [6] |
| **Brand** | 0.117  (0.103)  [17] | 0.305***  (0.0754)  [17] | 0.424***  (0.125)  [27] |
| **Number of Services provided** | -0.0473***  (0.0133) | -0.00319  (0.0181) | -0.0399**  (0.0146) |
| **Observations** | 593 | 267 | 334 |

Notes: Reference ownership type is NHS provider in all models. Coefficients from linear regression of overall quality rating scored as “Outstanding”=4, “Good”=3 ”Requires Improvement”=2 “Inadequate =1”. Robust Standard errors in parentheses. * p<0.05, ** p<0.01, *** p<0.001. The number of hospitals in each category are denoted by square brackets.

**References**

1. Care Quality Commission (CQC). The five key questions we ask. 2022. <https://www.cqc.org.uk/about-us/how-we-do-our-job/five-key-questions-we-ask>
2. Moscelli G, Gravelle H, Siciliani L. The effect of hospital choice and competition on inequalities in waiting times. Journal of Economic Behavior & Organization 2023;205:169–201.
